# Synchrotron X-ray tomography and spectroscopy in numismatics: disclosing counterfeit practices in medieval silver coins

**DOI:** 10.1107/S1600577526005771

**Published:** 2026-06-26

**Authors:** Simona Raneri, Alessandra Gianoncelli, Andrew King, Monica Baldassarri, Vincenzo Palleschi

**Affiliations:** a Department of Earth Sciences University of Florence Florence Italy; b Elettra Synchrotron, Trieste, Italy; c Synchrotron Soleil Saint-Aubin France; d Department of Historical Studies ’Federico Chabod’ University of Milan Milan Italy; e Institute of Chemistry and Organometallic Compounds Italian National Research Council Pisa Italy; SESAME, Jordan

**Keywords:** ancient coins, forgery detection, mercury traces, micro-computed tomography, micro-X-ray diffraction, micro-X-ray fluorescence

## Abstract

This study explores the use of advanced synchrotron radiation (SR)-based imaging and spectroscopic techniques to investigate the distribution and nature of mercury (Hg) in numismatic artifacts, with a particular focus on its role in historical counterfeiting practices. A selection of medieval silver coins exhibiting anomalous surface Hg signals, initially identified through portable energy-dispersive X-ray fluorescence (ED-XRF) and laser-induced breakdown spectroscopy, were further examined using high-resolution synchrotron X-ray micro-computed tomography (SR-µCT), micro-X-ray spectroscopy (SR-µXRF) and micro-X-ray diffraction (SR-µXRD) at the PSICHÉ beamline of the SOLEIL Synchrotron. Comparative analyses were conducted on coins produced by an unofficial mint at the Castle of Godano (La Spezia, Italy) and on verified genuine specimens. The results provide evidence for the use of Hg–Ag amalgam, indicative of fraudulent minting practices. This research highlights the methodological strength of integrating multiple non-destructive techniques. Portable ED-XRF demonstrates significant utility as a preliminary screening tool for detecting Hg in medieval and Renaissance coins, supporting the identification of potential forgeries. The combined use of SR-µCT, SR-µXRF and SR-µXRD offers a comprehensive framework for probing internal microstructures and elemental compositions of metal archaeological objects, advancing our understanding of both ancient coinage and fraudulent processes.

## Abstract 2

Advanced synchrotron-radiation-based imaging and spectroscopic techniques were used to investigate the distribution and nature of mercury in numismatic artifacts, with a particular focus on its role in historical counterfeiting practices.

## 1. Introduction

The compositional analysis of ancient coins is of paramount importance for the study of the historical and economic context in which they were produced and circulated. To this extent, the employment of non-destructive methodologies has become increasingly prevalent in the field of numismatic research in view of safeguarding these valuable artifacts while simultaneously facilitating a deeper understanding of their composition, manufacturing processes and historical context.

One of the earliest and most widely used non-destructive techniques in the field of numismatics is X-ray fluorescence (XRF), which has been employed for a variety of applications, ranging from elemental analysis and corrosion studies to conservation efforts (Brocchieri *et al.*, 2024 ▸; Corsi *et al.*, 2018 ▸; Torrisi *et al.*, 2013 ▸; Navas *et al.*, 2016 ▸; Scuotto *et al.*, 2014 ▸; Pagano *et al.*, 2023 ▸; Volpi *et al.*, 2023 ▸). In addition to XRF, laser-induced breakdown spectroscopy (LIBS) is growing in popularity within numismatic studies due to its minimally invasive characteristics and its ability to provide qualitative and quantitative information that can support compositional and contextual analyses (Gaudiuso *et al.*, 2019 ▸; Orlić Bachler *et al.*, 2016 ▸). Furthermore, LIBS can also be used to analyse the corrosion products or patina of historical objects (Pardini *et al.*, 2012 ▸). Also, X-ray computed microtomography has been demonstrated to be effective in the identification of coins, especially when affected by severe corrosion and encrustations that obscure surface details (Abate *et al.*, 2024 ▸).

Among non-conventional sources and non-destructive techniques, synchrotron methods have emerged as powerful tools for characterizing the surface and internal composition of ancient coins without compromising their integrity. In this context, synchrotron radiation XRF (SR-XRF) has been utilized and has been demonstrated to be highly valuable for the detailed analysis of alloys’ composition (Carlomagno *et al.*, 2022 ▸), manufacturing methods (Oudbashı *et al.*, 2017 ▸), provenance determination of metallic artifacts (Vasilescu *et al.*, 2010 ▸) and characterization of surface corrosion products (Rodrigues *et al.*, 2012 ▸). In addition to SR-XRF, other synchrotron-based techniques, such as micro-X-ray diffraction (µXRD), have been demonstrated to be effective in the analysis of ancient coins for visualizing and interpreting features obscured by corrosion while characterizing corrosion products themselves (Adriaens *et al.*, 2010 ▸).

Apart from alloy characterization, understanding manufacturing methods and the study of corrosion products, a further challenge within numismatic studies is the identification of counterfeit coins. Counterfeiting has a long history dating back to Roman times, when the production and circulation of counterfeit coinage was widespread across the Empire and beyond its borders (Dymowski & Myzgin, 2024 ▸; Higbie, 2023 ▸). Archaeological and historical methods have been used to study the composition of Roman and medieval European coins; in some cases, these studies have shown the existence of silver (Ag) coins with an anomalous enrichment of silver in a thin surface layer on a tundish made from vile metal, typically copper, brass or bronze (Ingo *et al.*, 2017 ▸; Pardini *et al.*, 2012 ▸; Arias *et al.*, 2017 ▸). In metallurgical terms, brass indicates Cu–Zn alloys, while bronze refers to Cu–Sn–based systems, often including ternary alloy Cu–Sn–Pb finalized to improve bronze castability. The compositional ranges of these alloys in historical coinage are highly variable, typically spanning from a few to several tens of atomic percent of the alloying element, depending on the technological context and intended properties; such variations influence phase constitution, mechanical behaviour and corrosion patterns (Scott, 1991 ▸). Vlachou-Mogire *et al.* (2007 ▸) presented an overview of the hypotheses suggested in the past to understand the technical procedures used for manufacturing silver plating coins (Vlachou-Mogire *et al.*, 2007). While a consensus has been reached on the technique used for plating Roman Republican denarii (third–first century BCE), namely applying a thin silver lamina to the hot tundish before coinage (this practice is recognized as counterfeiting), several hypotheses have been suggested for Late Roman and medieval coinage. Most recent studies on the topic seem to agree on the use of amalgam silvering. From a materials science perspective, silver amalgam consists of an Hg–Ag system in which mercury (Hg) acts as a solvent for silver; upon heating, Hg evaporates, leaving an Ag-rich layer on a metal tundish whose residual Hg traces depend on the initial composition and the thermal treatment.

Although mercury-based silvering techniques were known in China as early as the first century BCE (La Niece, 1990 ▸), there is no clear evidence of their use in Europe prior to the Middle Ages. Vlachou-Mogire *et al.* (2007 ▸) highlight the scarcity of references to amalgam silvering in historical texts, in contrast to the more widely documented amalgam gilding. The limited distinction between the two processes may explain this, as medieval sources such as Theophilus and Biringuccio describe only minor differences – primarily in mercury proportions and cooling methods (Theophilus, 1963 ▸; Biringuccio, 1959 ▸). Notably, a Byzantine manuscript deviates from Theophilus’s guidelines by recommending the same mercury ratio for silvering as for gilding, while adopting Biringuccio’s method of oil immersion followed by heating to complete the process, instead of the traditional urine quenching used in gilding. Evidence for the presence of mercury on ancient coins has been documented through various analytical approaches. Vlachou-Mogire *et al.* (2007 ▸) detected mercury using micro-destructive laser-ablation inductively coupled plasma-mass spectrometry (LA-ICP-MS) on Late Roman coins (293/4–305 CE) from 13 mints, housed in the Cope’s archive at the British Museum. They linked mercury to a thin (∼1 µm) silver layer over a Cu–Sn–Pb–Ag alloy, suggesting amalgam silvering was an official minting technique introduced in response to Gallienus’ debasement (reducing silver to ∼2%) and possibly continued under Diocletian’s reforms. Further support for official amalgam silvering comes from Romano *et al.* (2012 ▸), who identified mercury on the surfaces of Roman nummi (320–333 CE) from the Misurata Treasure (Libya) using non-destructive XRF. Mercury was notably associated with coins from specific mints – Rome, Constantinople, Ticinum and Aquileia – during a limited time period. In contrast, Uhlir *et al.* (2016 ▸) and Gaudiuso *et al.* (2019 ▸) observed mercury on sixth–seventh century Sasanian coins, proposing post-mint contamination from medicinal mercury-based poultices. Their analysis, XRF on sections and LIBS, showed silver depletion in the mercury-containing surface layers, ruling out silver amalgam use since both the XRF analysis on coin sections (destructive) and the LIBS (micro-destructive) analysis showed depletion of silver in the mercury layer at the surface. Notably, mercury was found on 152 of 188 coins from the free market, but only three of 40 from the Kunsthistorisches Museum, which they attributed to either cleaning restoration practices or alternate purposes such as aesthetic enhancement. On the other hand, several other studies have correlated the use of silver amalgam in coins to counterfeiting. Beck *et al.* (2017 ▸) found that fake coins from the Preuschdorf treasure in France were made using an amalgam of mercury and silver, with 14 out of 7327 of the identified fake coins containing this mixture; these fake coins date back to the late 16th and early 17th centuries.

In this context, recent historical and archaeological investigations at Godano Castle – an important fortress on the border between Liguria and Tuscany in northern Italy – have provided clear evidence of clandestine minting activity (Baldassarri *et al.*, 2018 ▸). Built between the 12th and 13th centuries, the castle was initially controlled by the lords of Godano and later by the Malaspina family, until its destruction in 1524. Excavations have uncovered a range of mint-related materials, including copper-alloy sheets, flattened strips, casting sprues, lead ingots, smooth coin blanks and a number of counterfeit coins, confirming the operation of an unofficial mint within the castle. This mint appears to have produced imitations of Genoese, Piedmontese, Tuscan and Bolognese coins. The detection of mercury on some of the counterfeit silver coins recovered from the site – dated to the 15th–16th centuries – reopens the question of the use of mercury-based silvering techniques in historical coin forgery in Europe, a topic previously supported by only a few isolated cases (such as a ninth–tenth century Iranian dirham, a 13th century fourpence and an unidentified 15th century coin).

In the current study by means of µCT combined with XRF and XRD techniques, we analysed coins produced by the unofficial mint at the Castle of Godano and compared them with a counterfeit suberate Roman republican denarius and with two verified genuine specimens from archaeological sites.

These findings provide new data for re-evaluating the extent and chronology of such practices and strategies for detecting it using integrated and advanced analytical approaches.

## 2. Materials and methods

### 2.1. The counterfeit coins from Godano

**Figure 1:**
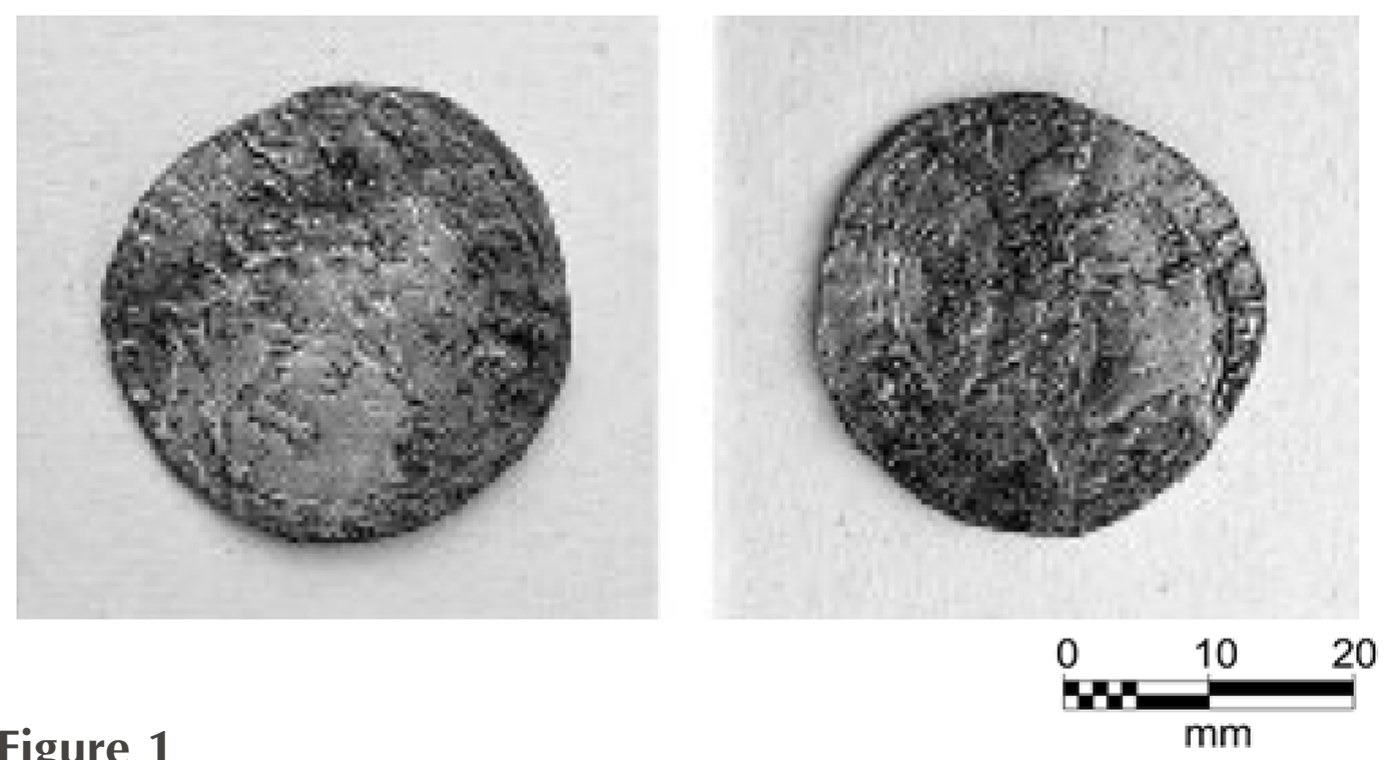
The 5-soldi grosso of Carmagnola mint (Godano counterfeit).

Among the various counterfeit coins found at Godano, two in particular were selected for the present study, as they have proven especially interesting: one is an imitation of a 5-soldi grosso from the Carmagnola mint, originally struck between 1504 and 1528; the other is a quattrino from Siena, likely dating between 1487 and 1507 (Baldassarri *et al.*, 2025 ▸). For comparison, two additional coins not from the archaeological site were also analysed: an authentic Sienese quattrino minted between 1504 and 1507 (bearing the mark of the mint master Francesco Castoro), and a suberate Roman republican denarius, contemporary counterfeit of a coin original issued by L. Valerius Flaccus, dated to 108–107 BCE (Figs. 1 ▸, 2 ▸ and 3 ▸) (Baldassarri *et al.*, 2025 ▸).

### 2.2. Preliminary XRF and LIBS laboratory analysis

The counterfeit coins from the clandestine mint at Godano were initially analysed using energy-dispersive X-ray fluorescence (ED-XRF) and LIBS. The XRF spectra were collected with an acquisition time of 90 s with the Elio (Bruker) instrument, a portable spectrometer operated at a voltage of 40 kV and a current of 80 µA (320 mW), with 1 mm^2^ spot size. LIBS analysis was performed using the Modì instrument in stratigraphy modality. Modì is a double-pulse LIBS instrument developed by CNR (Bertolini *et al.*, 2006 ▸). Two collinear laser beams (40 and 60 mJ, in pulses lasting 20 ns) were focused on the surface of the sample using a lens with a focal length of 100 mm. The delay between the two pulses was set to 1 µs. The light emitted by the plasma generated by the second laser pulse was collected with an optical fibre, placed at a 45° angle to the sample surface and ∼1 cm from the analysis point. The plasma emission thus collected was analysed using an Avaspect USB-2 spectrometer (Avantes) to reconstruct the LIBS emission spectrum between 200 and 900 nm, with a resolution of 0.1 nm between 200 and 430 nm and of 0.3 nm between 430 nm and 900 nm. Twenty laser pulses were acquired in sequence, in the same position on the surface of the coin. Each laser pulse ablates a minimal part of the sample, so that different depths under the surface can be explored, giving information about the stratigraphic composition of the sample.

### 2.3. X-ray tomography, fluorescence and diffraction at the PSICHÉ beamline (SOLEIL Synchrotron)

The PSICHÉ beamline (Pression Structure Imagerie par Contraste à Haute Énergie) at the SOLEIL Synchrotron is specifically designed for conducting XRD studies under extreme pressure and temperature conditions, as well as for performing high-energy X-ray tomography (up to 120 keV). SR-µCT was carried out using a Hamamatsu ORCA Flash4.0 camera, paired with a 250 µm-thick LuAG scintillator. This setup employed two Hasselblad 100 mm lenses in a tandem configuration, resulting in an effective pixel resolution of 6 µm (Mittone *et al.*, 2017 ▸). Additionally, spatially resolved energy-dispersive X-ray diffraction (ED-XRD), as described by King *et al.* (2019 ▸), enables SR-µXRD and micro-X-ray spectroscopy (SR-µXRF) analyses of the samples.

To advance the study of medieval counterfeiting practices, tomographies were acquired with the aim of reconstructing the full volume of the coins. A half-acquisition protocol using an offset rotation axis enabled the investigation of volumes of ∼20 mm × 20 mm × 3 mm in a single scan, limited to 3 mm by the height of the beam. A helicoidal acquisition was used to extend the field of view in the vertical direction. Given the large diameter of the coins (>10 mm), signal attenuation becomes significant when the coin’s plane is nearly parallel to the beam. Acquisition parameters were adjusted to use the full dynamic range of the camera in order to obtain as much exploitable radiographic data as possible. Although some limited-angle and beam-hardening artefacts remain, the tomographic images provide valuable insights into the distribution of silver and mercury across the coin thickness. Variations in the local attenuation coefficient correspond to differences in elemental composition, enabling a more detailed characterization of the Hg and Ag layers.

To confirm which elements are present, and where, in the coins, tomographic reconstructions were used to select regions of interest for measurement by XRF and XRD. We worked at the highest available energies of the PSICHÉ beamline using a polychromatic spectrum with photon energies 30 and 100 keV and a beam size of 30 µm × 30 µm for the incoming beam and for the detector slits. Both diffraction peaks and fluorescence peaks are observed with the same energy-sensitive germanium detector. The ED diffraction geometry uses two sets of slits to define both the incident and diffracted beams. The observed signal comes from the gauge volume defined by the intersection of the two beams (confocal geometry). The spatial resolution depends on the size of the beams and the diffraction angle. The Bragg formula, in the fixed-angle ED geometry used in PSICHÉ, links the energy (*E*) of the peaks with the reticular *d* spacing (*d*) of the Cu, Sn and Ag corrosion products on the coin surface in the form
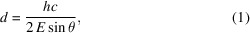
where 2θ is the fixed angle between the diffracted beam and the SR beam, *h* is Planck’s constant, and *c* is the speed of light. XRF peaks are observed at their characteristic energies.

**Figure 4:**
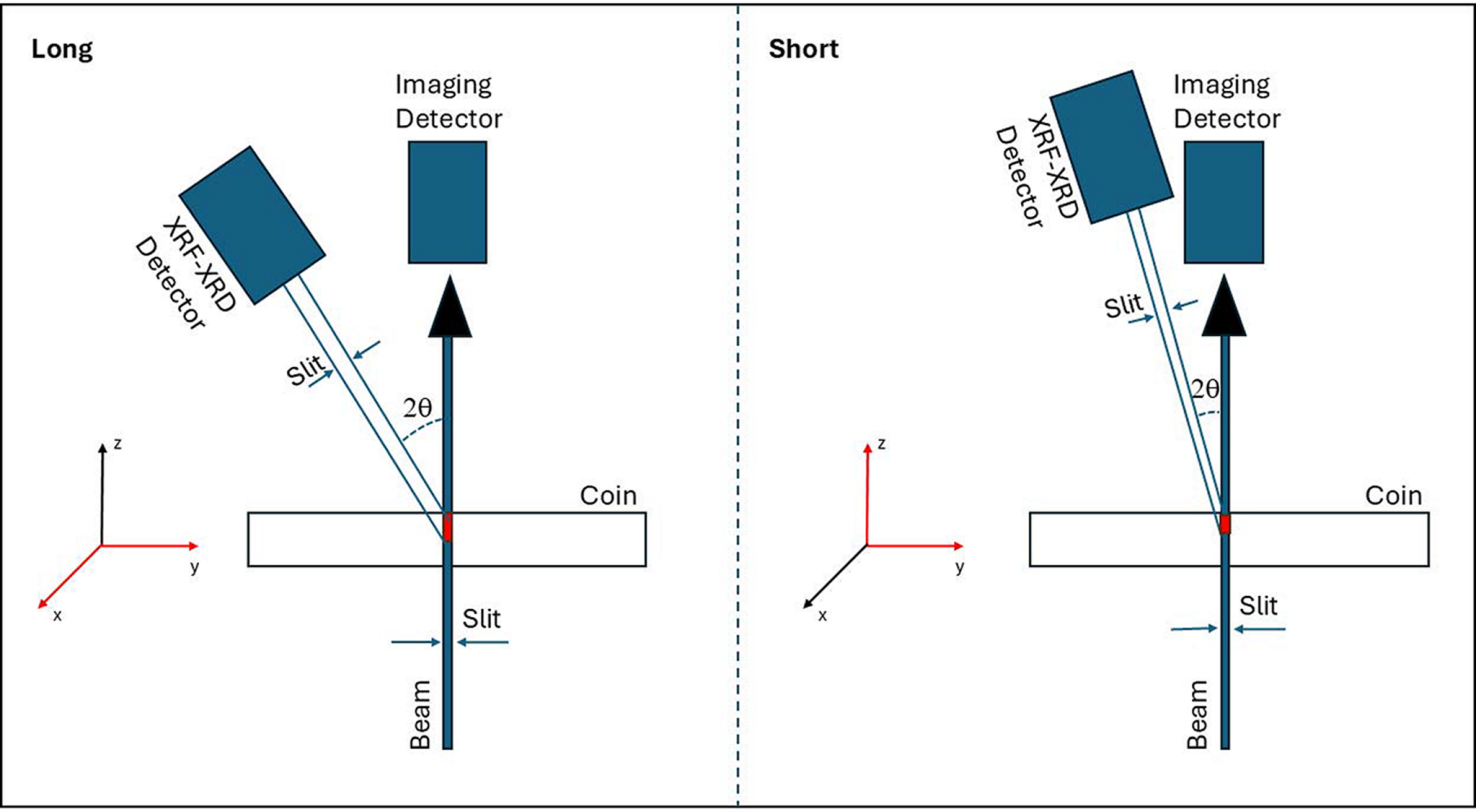
Experimental configuration for tomography, XRF and XRD at PSICHÉ.

We worked at two diffraction angles (2θ = 8° and 2θ = 30°) and with different experimental configurations for reconstructing both depth-averaged maps of the coin surface (‘long’ configuration) and ‘virtual’ cross sections (‘short’ configuration) (see Fig. 4 ▸).

## 3. Results and discussion

### 3.1. ED-XRF and LIBS analysis

**Figure 5:**
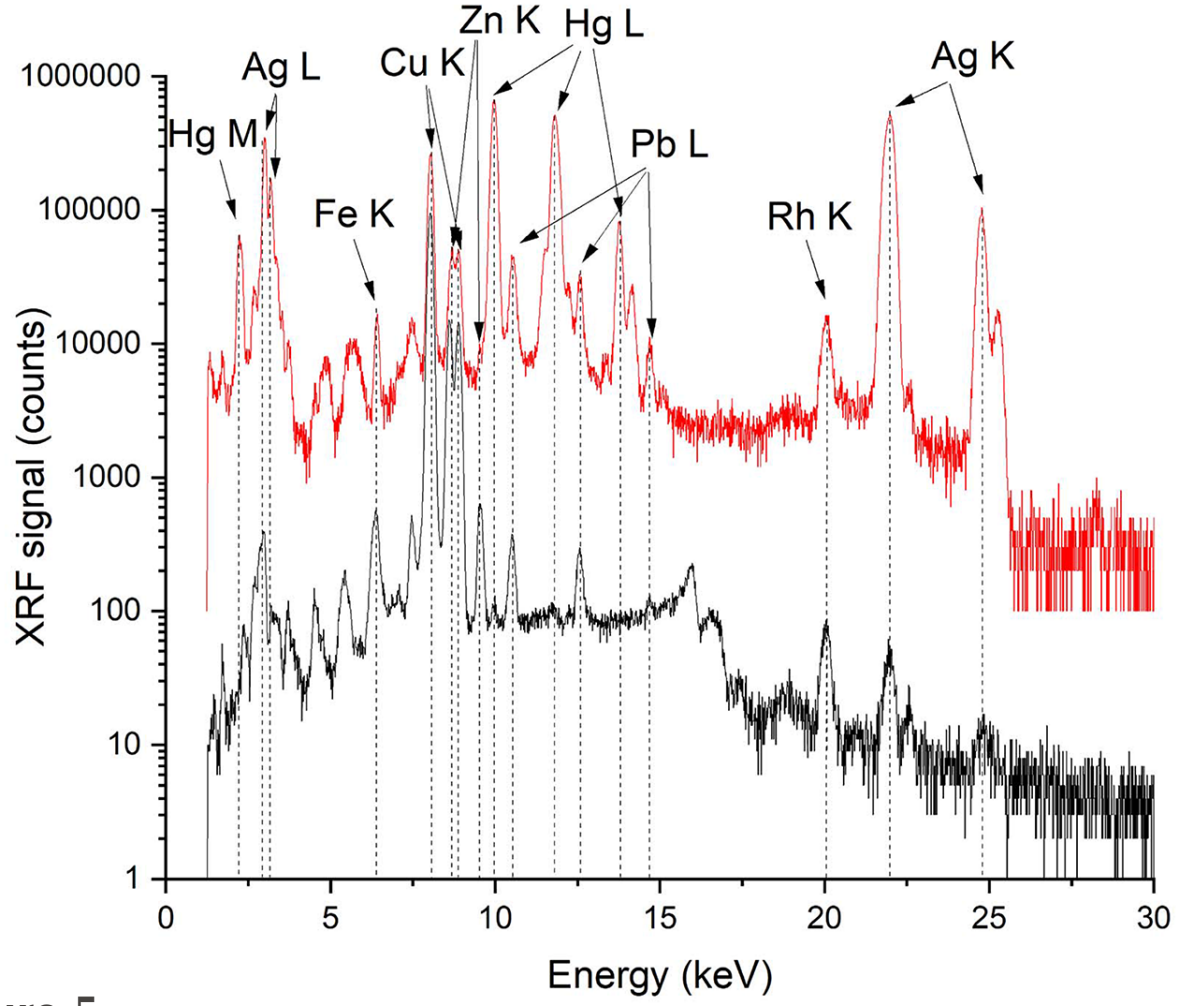
Black: XRF spectrum on a region of the coin without silvering. Red: XRF spectrum on a region of the coin with silvering. Note the coexistence of Hg and Ag emission at the coin surface and brass (Cu + Zn) from the underlying tundish. The *y*-axis is logarithmic, the curves are vertically shifted for easing the comparison.

Preliminary ED-XRF analyses of the silver coins from the unofficial mint revealed the presence of mercury localized at the coin surface, colocalized with silver (Fig. 5 ▸). Although the exact origin of mercury remains to be fully elucidated, its correlation with counterfeiting strongly suggests the utilization of a mercury–silver amalgam during the production process.

The depth profile of Hg and Ag was estimated from LIBS intensity, which is approximately proportional to elemental concentration. To correct for geometrical factors that may attenuate signal intensity with increasing depth, Hg and Ag signals were normalized to the Cu signal. The choice of copper as an internal standard is supported by its relatively light atomic mass and its diffusion into the amalgam over centuries, resulting in an almost uniform distribution within the amalgam layer.

**Figure 6:**
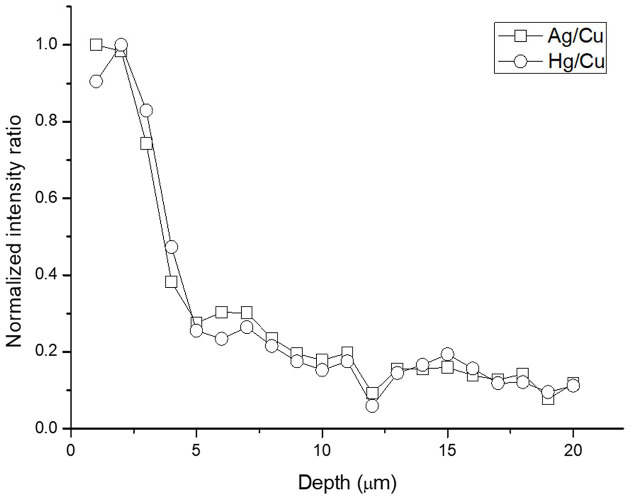
In-depth analysis of Ag and Hg distribution below the grosso of Carmagnola sample’s surface.

LIBS studies of the Godano coins suggest the coexistence of mercury and silver in a thin Hg–Ag amalgam layer, generally thinner than 5 µm (Fig. 6 ▸), deposited over a copper or brass substrate. However, the micro-destructive nature of LIBS limits the precise determination of mercury distribution, especially through the thickness of the coin. Additionally, some coins are extremely thin and lack visible layering, further complicating the analysis.

### 3.2. X-ray tomography

**Figure 7:**
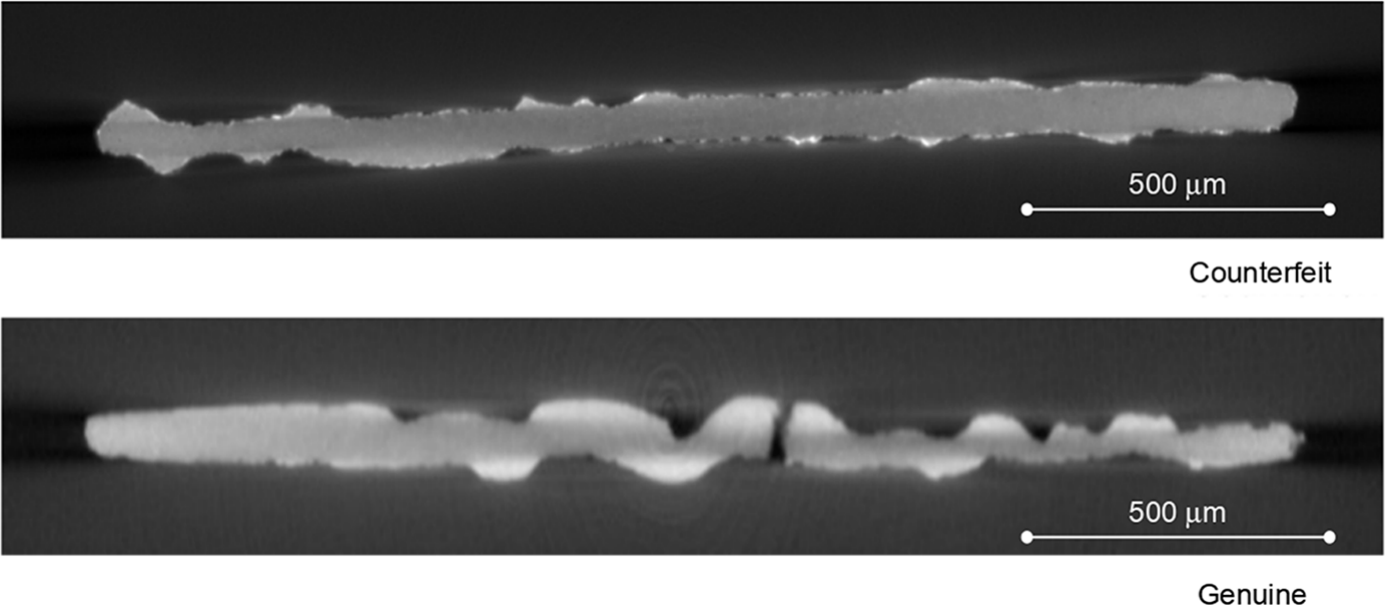
Comparison between tomographic images of the counterfeit (top) and genuine (bottom) quattrino of Siena selected as representative of the dataset.

Fig. 7 ▸ presents reconstructed tomographic cross sections selected as representative of the dataset of a counterfeit quattrino from Siena alongside a genuine specimen, both recovered from the Godano Castle site. The images reveal a very thin surface layer on the counterfeit coin, characterized by regions of markedly higher attenuation coefficients (white voxels) compared with the underlying structure. By contrast, the genuine coin (Fig. 7 ▸, bottom panel) displays a relatively homogeneous attenuation profile throughout its cross section, aside from minor artefacts related to limited-angle acquisition and beam hardening. This localized high-attenuation surface layer on the counterfeit coin aligns with the detection of mercury on its surface and is consistent with the presence of an Hg–Ag amalgam, which was absent in the genuine counterpart.

**Figure 8:**
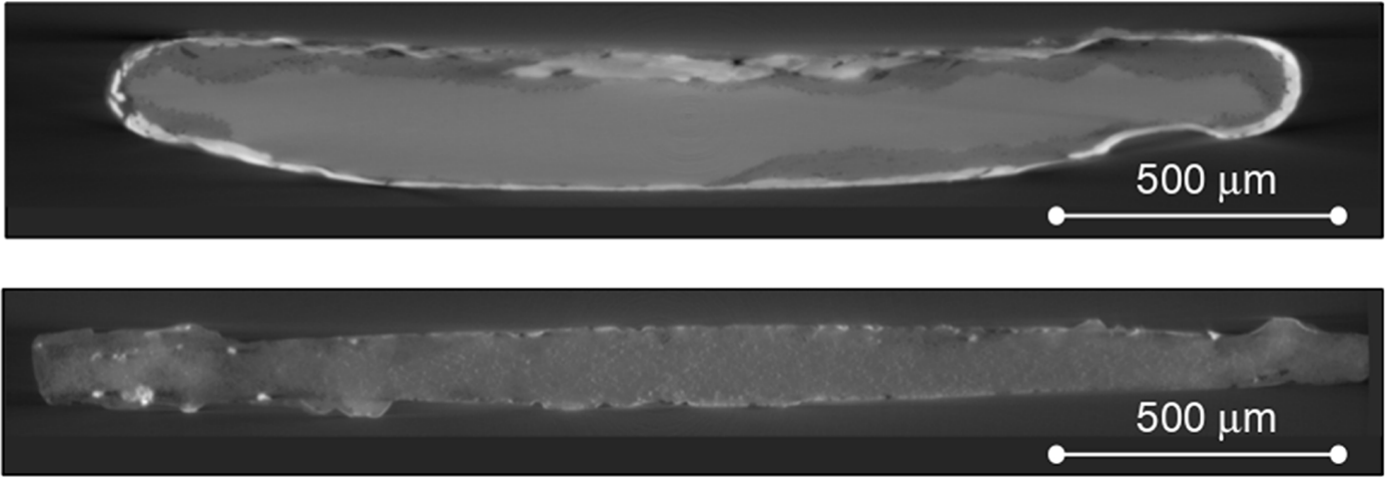
Comparison between tomographic images of the Roman suberate denarius (top) and the counterfeit 5-soldi grosso of Carmagnola (bottom) selected as representative of the dataset.

Fig. 8 ▸ compares tomographic cross sections selected as representative of the dataset of a Roman suberate denarius and a counterfeit grosso from Carmagnola, illustrating distinct differences in surface layering. The denarius exhibits a relatively thick uniform surface layer (∼30µm) with consistent attenuation values, indicative of a homogeneous coating. *In situ* spectroscopic analyses confirmed the presence of silver in this layer, consistent with the lamina silvering technique documented in historical sources. In contrast, the grosso shows a thinner discontinuous surface layer with variable attenuation. Complementary XRF and LIBS analyses detected both silver and mercury within this layer, supporting the interpretation of a mercury–silver amalgam surface treatment also in this case.

### 3.3. Detecting mercury at the PSICHÉ beamline

**Figure 9:**
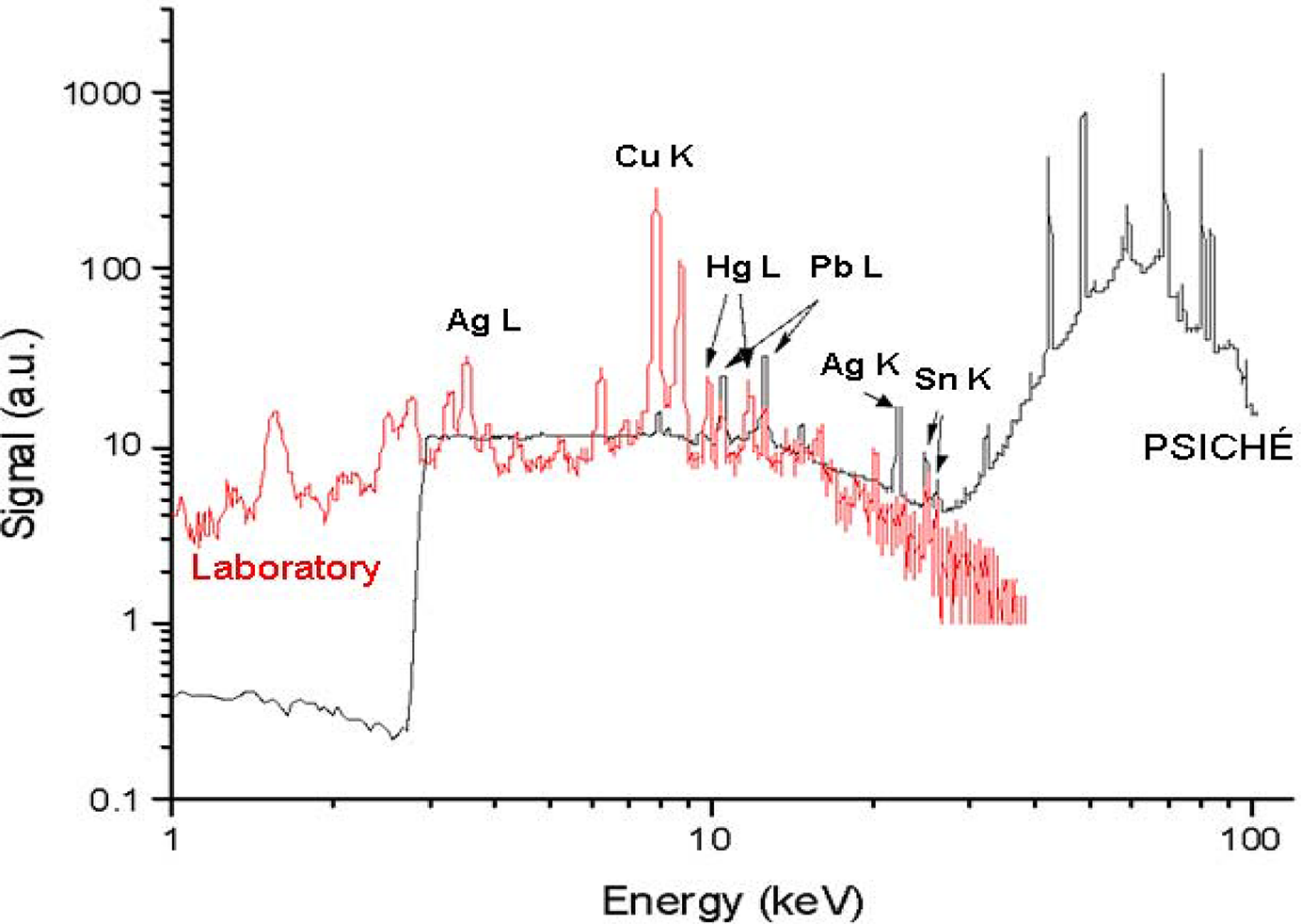
Comparison between the XRF signal obtained in the laboratory (red curve) and the XRF–XRD spectrum at PSICHÉ. The sample is the counterfeit quattrino of Siena from Godano castle. Note the logarithmic scale on both *x* and *y* axes.

Due to the X-ray tube excitation energy and to the silicon drift detector thickness, the laboratory XRF instrument allows for an efficient detection until 15 keV, thus showing Cu *K* lines, Hg *L* lines and Ag *L* lines (red spectrum in Fig. 9 ▸). The use of an excitation higher energy and a Ge detector at the PSICHÉ beamline better highlights emission peaks with energy above 10 keV, and thus Ag and Sn *K* lines and Pb *L* lines, probing also a deeper layer in the samples (black spectrum in Fig. 9 ▸).

At the PSICHÉ beamline, both silver (Ag) and mercury (Hg) were identified through their characteristic diffraction and fluorescence signals.

Mercury detection relied on its XRF emission lines, specifically the *K* lines at 70.82 and 80.25 keV and the *L* lines at 9.99 and 11.82 keV. By operating at a 30° angle, a spatial resolution of ∼30 µm × 30 µm × 50 µm was achieved. Silver was detected via XRF lines at 22.2 and 24.9 keV, as well as diffraction peaks between the {311} and {420} reflections, observed in the 70–100 keV range at a diffraction angle (2θ) of 8°. Under these conditions, the diffraction signal was collected from a gauge volume roughly 30 µm × 30 µm × 175 µm, with the long axis oriented along the beam, effectively averaging over a surface layer.

**Figure 10:**
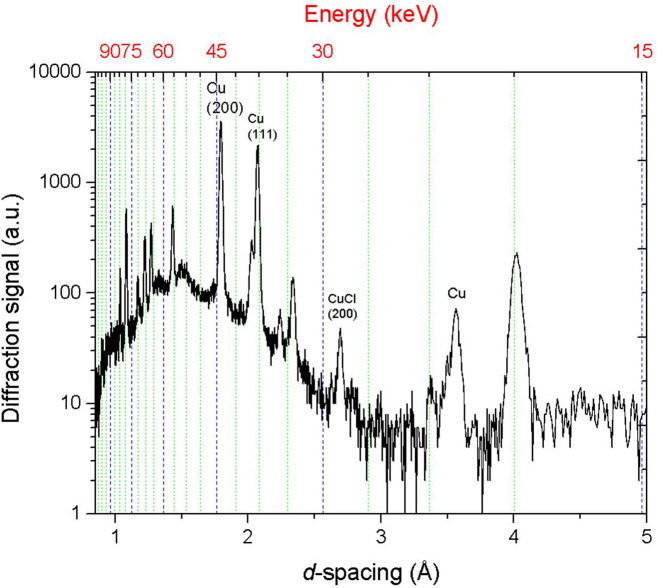
Equivalence between *d*-spacing parameter and XRF–XRD energy (counterfeit quattrino of Siena). Some diffraction lines are identified. Note the logarithmic scale on both upper-*x* and *y* axes.

The brass tundish beneath the Hg–Ag layer was characterized primarily by the Sn *K* lines at 25.3 and 28.5 keV. Copper (Cu) and zinc (Zn) *K* lines were scarcely visible due to the detectors’ reduced sensitivity below 10 keV; additionally, many diffraction peaks associated with copper and its corrosion products are well visible in the XRF–XRD spectrum (Fig. 10 ▸).

### 3.4. Surface maps and virtual cross sections of the coins

**Figure 11:**
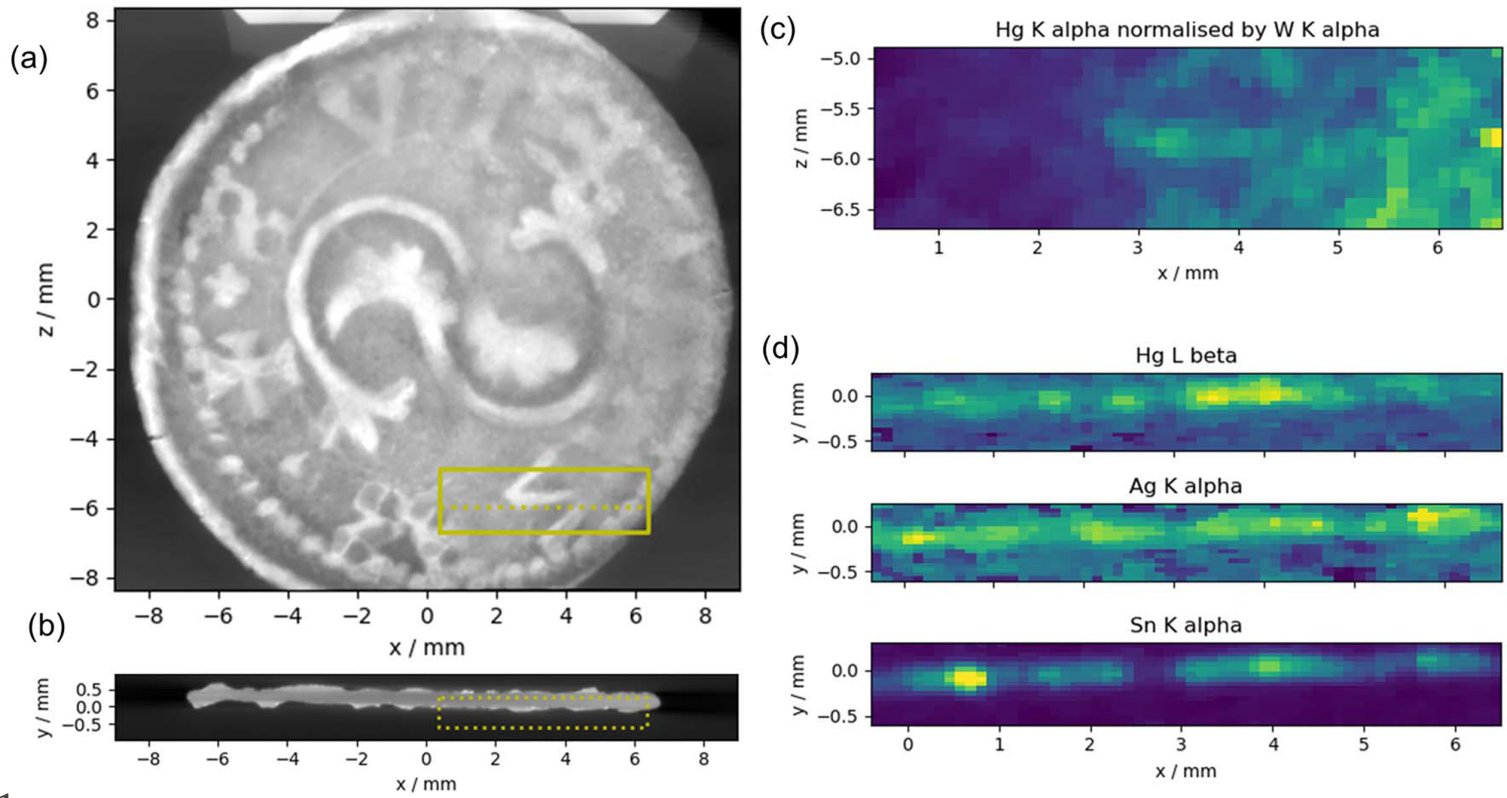
The counterfeit quattrino of Siena from the unofficial mint of Godano. Average value projection from tomography data, compared with fluorescence mapping data. (*a*) Projection of one face from the tomography data. (*b*) Cross section through the same dataset. (*c*) 2D surface map of the mercury XRF signal. (*d*) Cross-section XRF maps of mercury, silver and tin. The areas corresponding to the XRF maps are marked in yellow on the tomography images. In the scan made in the section of the coin we observe correlated Hg and Ag signals from the surface.

At the PSICHÉ beamline, working at two diffraction angles (2θ = 8° and 2θ = 30°) and using different experimental configurations, we reconstructed both depth-averaged surface maps and virtual cross sections of the coins [Figs 11(*a*) and 11(*b*)]. The surface maps, which represent elemental distributions averaged over the coin’s thickness, enable visualization of the microscopic surface distribution of Ag, Sn, Cu and Hg (at both 2θ = 8° and 30°) [Fig. 11 ▸(*c*) ▸].

In contrast, the virtual cross sections obtained at 2θ = 8° provide information on the in-depth distribution of silver, tin and copper beneath the coin surface. The virtual cross section of the counterfeit quattrino of Siena along a line at the coin’s surface [Fig. 11(*d*) ▸] shows the correlation between the XRF signals of silver and mercury.

Despite the difficulty of acquiring the Hg signal, due to the interferences with the Cu XRD signal, which prevents the use of the high-energy Hg *K* lines, and the poor sensitivity of the detector at the energies of its *L* lines, both the surface maps and the virtual XRF sections confirm the coexistence of Ag and Hg on the counterfeit coins from Godano. The results are consistent with the presence of a single surface layer created through the cold deposition of an Hg–Ag amalgam, followed by heating directly on the base metal substrate to allow the mercury to evaporate, leaving a thin layer of silver on the coin’s surface.

## 4. Conclusions

The combined X-ray tomographic and XRF/XRD analyses performed at the PSICHÉ beamline have yielded valuable insights into the counterfeiting techniques employed by forgers at Godano. Specifically, the identification of a silver–mercury amalgam used to apply a thin silver layer onto a base metal planchet reveals a falsification method that differs markedly from those documented in Roman-era coinage. This finding expands our understanding of medieval and Renaissance numismatic forgery practices.

From a methodological perspective, the detection of mercury on counterfeit coins demonstrates the effectiveness of non-destructive, accessible and portable ED-XRF instrumentation as a preliminary screening tool. Mercury presence can thus serve as a practical indicator of forgery, facilitating the authentication process for medieval and Renaissance coins in both private collections and museum contexts.

## Figures and Tables

**Figure 2:**
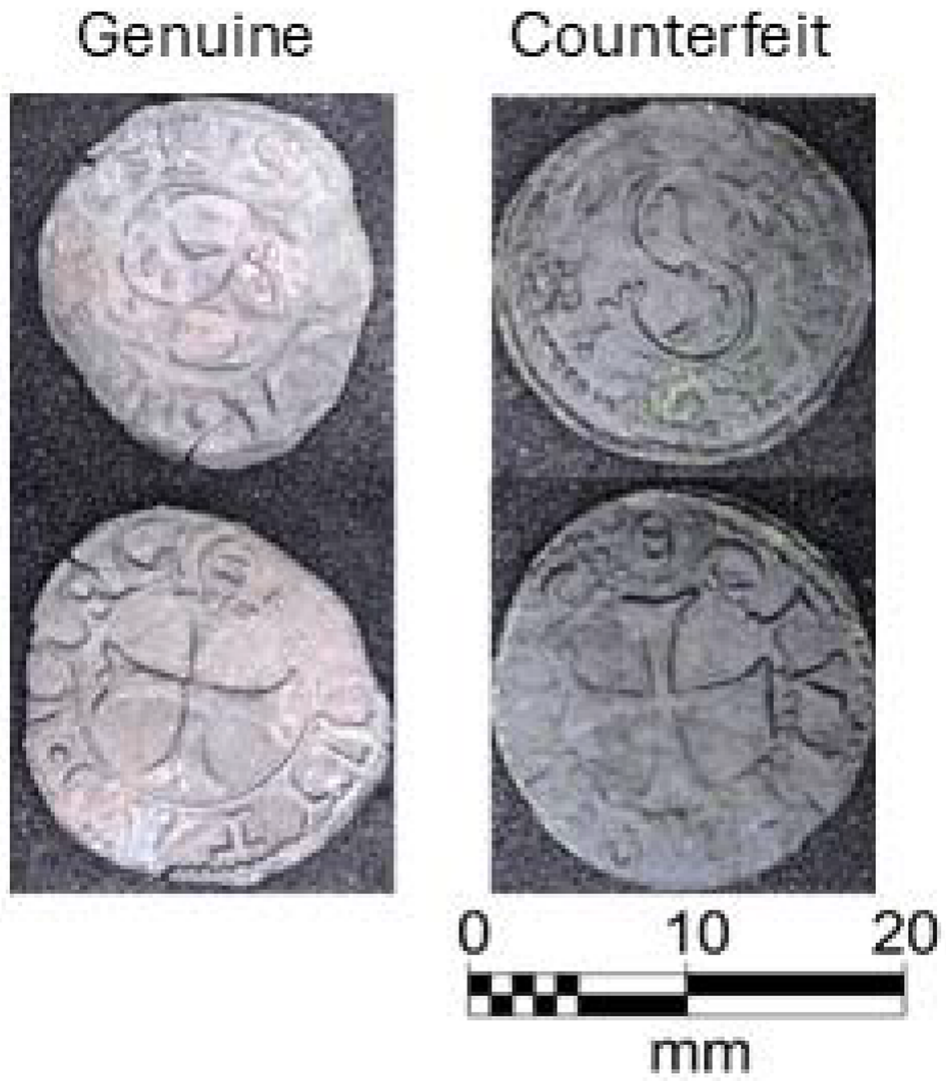
The two quattrini of Siena studied. At the left a genuine quattrino, at the right the counterfeit one found at the Godano site.

**Figure 3:**
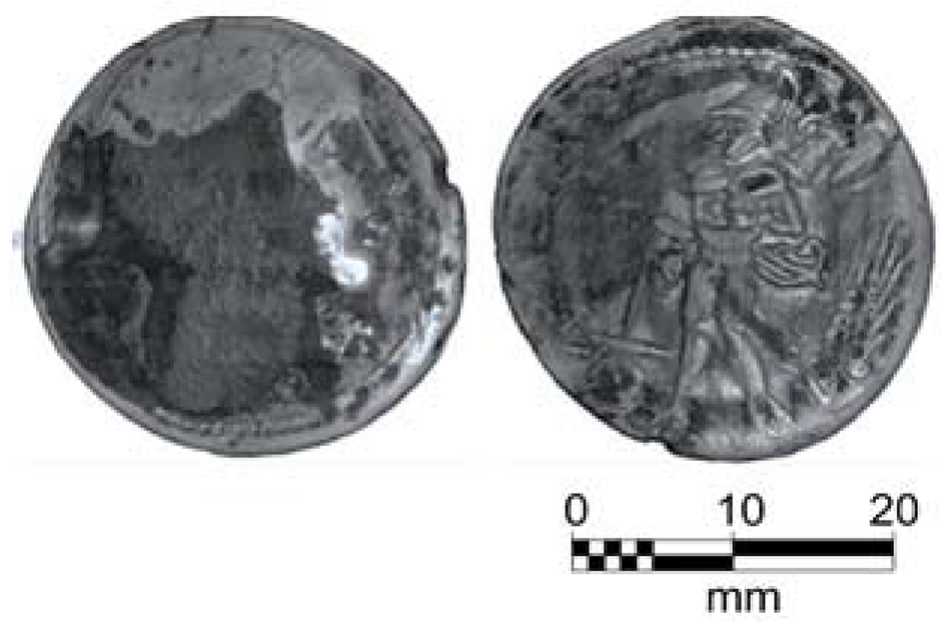
Suberate silver denarius (Valerius Flaccus Denarius, 108–107 BCE). Three-dimensional image collected at PSICHÉ beamline.

## Data Availability

Data are available upon reasonable request to the corresponding authors.

## References

[bb242] Abate, F., De Bernardin, M., Stratigaki, M., Franceschin, G., Albertin, F., Bettuzzi, M., Brancaccio, R., Bressan, A., Morigi, M. P., Daniele, S. & Traviglia, A. (2024). *J. Cult. Heritage*, **66**, 436–443.

[bb1] Adriaens, A., Dowsett, M., Lehmann, E., Farhi, Y., Gunneweg, J. & Bouchenoire, L. (2010). *Holistic Qumran – Trans-disciplinary Research of Qumran and the Dead Sea Scrolls*, pp. 11–20. Brill.

[bb2] Arias, C., Bani, S., Catalli, F., Lorenzetti, G., Grifoni, E., Legnaioli, S., Pagnotta, S. & Palleschi, V. (2017). *Appl. Spectrosc.* **71**, 817–822. 10.1177/0003702816641421 27154737

[bb3] Baldassarri, M., Chiarenza, N., Pagnotta, S., Palleschi, V., Parodi, L. & Salvatori, E. (2018). *AMediev.* **45**, 335–356.

[bb4] Baldassarri, M., Palleschi, V. & Raneri, S. (2025). *Una zecca di falsari nel castello di Godano (La Spezia): note sulla tecnica di produzione e di argentatura delle monete sullo scorcio nel medioevo*, pp. 85–108 of *Le molte facce di una moneta. Denaro e materialità nella Storia: saggi in onore di Lucia Travaini.* Milano University Press.

[bb5] Beck, L., Alloin, E., Vigneron, A., Caffy, I. & Klein, U. (2017). *Nucl. Instrum. Methods Phys. Res. B* **406**, 93–98.

[bb6] Bertolini, A., Carelli, G., Francesconi, F., Francesconi, M., Marchesini, L., Marsili, P., Sorrentino, F., Cristoforetti, G., Legnaioli, S., Palleschi, V., Pardini, L. & Salvetti, A. (2006). *Anal. Bioanal. Chem.* **385**, 240–247. 10.1007/s00216-006-0413-6 16614821

[bb7] Biringuccio, V. (1959). *The Pirotechnica of Vannoccio Biringuccio.* New York: American Institute of Mining and Metallurgical Engineers.

[bb8] Brocchieri, J., Vitale, R. & Sabbarese, C. (2024). *X-ray Spectrom.* **53**, 452–463.

[bb9] Carlomagno, I., Zeller, P., Amati, M., Aquilanti, G., Prenesti, E., Marussi, G., Crosera, M. & Adami, G. (2022). *Sci. Rep.* **12**, 15919. 10.1038/s41598-022-19682-8 PMC9508250 36151121

[bb10] Corsi, J., Lo Giudice, A., Re, A., Agostino, A. & Barello, F. (2018). *Archaeol. Anthropol. Sci.* **10**, 431–438.

[bb244] Dymowski, A. & Myzgin, K. (2024). Editors. *Counterfeits, imitations, and copies of Roman Imperial denarii: making and faking coins on both sides of the limes*. Brepols.

[bb11] Gaudiuso, R., Uhlir, K. & Griesser, M. (2019). *J. Anal. At. Spectrom.* **34**, 2261–2272.

[bb246] Higbie, C. (2023). *The Fluidity of False Coins. In Forgery Beyond Deceit: Fabrication, Value, and the Desire for Ancient Rome.* Oxford University Press.

[bb12] Ingo, G. M., Riccucci, C., Faraldi, F., Pascucci, M., Messina, E., Fierro, G. & Di Carlo, G. (2017). *Appl. Surf. Sci.* **421**, 109–119.

[bb13] King, A., Guignot, N., Deslandes, J.-P., Pelerin, M., Joosten, I., De Looff, D., Li, J., Bertrand, L., Rosenberg, E., Dewaele, A., Boulard, E., Le Godec, Y., Perrillat, J.-P., Giovenco, E., Morard, G., Weitkamp, T., Scheel, M., Perrin, J., Chevreau, H. & Itié, J.-P. (2019). *Integr. Mater. Manuf. Innov.* **8**, 551–558.

[bb14] La Niece, S. (1990). *Antiq. J.* **70**, 102–114.

[bb15] Mittone, A., Manakov, I., Broche, L., Jarnias, C., Coan, P. & Bravin, A. (2017). *J. Synchrotron Rad.* **24**, 1226–1236. 10.1107/S160057751701222X 29091066

[bb111] Navas, M. J., Asuero, A. G. & Jiménez, A. M. (2016). *Appl. Spectrosc.* **70**, 207–221. 10.1177/0003702815616594 26767646

[bb16] Orlić Bachler, M. O., Bišćan, M., Kregar, Z., Jelovica Badovinac, I., Dobrinić, J. & Milošević, S. (2016). *Spectrochim. Acta B*, **123**, 163–170.

[bb17] Oudbashı, O., Hasanpour, A., Jahanpoor, A. & Rahjoo, Z. (2017). *Sci. Technol. Archaeol. Res.* **3**, 194–205.

[bb24] Pagano, S., Balassone, G., Germinario, C., Grifa, C., Izzo, F., Mercurio, M., Munzi, P., Pappalardo, L., Spagnoli, E., Verde, M. & De Bonis, A. (2023). *Heritage*, **6**, 2038–2055.

[bb18] Pardini, L., El Hassan, A., Ferretti, M., Foresta, A., Legnaioli, S., Lorenzetti, G., Nebbia, E., Catalli, F., Harith, M. A., Diaz Pace, D., Anabitarte Garcia, F., Scuotto, M. & Palleschi, V. (2012). *At. Spectrosc.* **74–75**, 156–161.

[bb20] Rodrigues, M., Schreiner, M., Melcher, M., Guerra, M., Salomon, J., Radtke, M., Alram, M. & Schindel, N. (2012). *X-ray Spectrom.* **41**, 416–424.

[bb21] Romano, F. P., Garraffo, S., Pappalardo, L. & Rizzo, F. (2012). *At. Spectrosc.* **73**, 13–19.

[bb22] Scott, D. A. (1991). *Metallography and Microstructure of Ancient and Historic Metals*, p. 123. Marina del Rey, CA: Getty Conservation Institute in association with Archetype Books.

[bb23] Scuotto, M., Bassi, C., Lezzerini, M., Grifoni, E., Legnaioli, S., Lorenzetti, G., Pagnotta, S. & Palleschi, V. (2014). *X-ray Spectrom.* **43**, 370–374.

[bb25] Theophilus (1963). *On Divers Arts: The Treatise of Theophilus.* The University of Chicago Press.

[bb26] Torrisi, L., Italiano, A., Cutroneo, M., Gentile, C. & Torrisi, A. (2013). *J. X-ray Sci. Technol.* **21**, 381–390. 10.3233/XST-130389 24004868

[bb27] Uhlir, K., Padilla-Alvarez, R., Migliori, A., Karydas, A. G., Božičević Mihalić, I., Jakšić, M., Zamboni, I., Lehmann, R., Stelter, M., Griesser, M., Schindel, N. & Alram, M. (2016). *Microchem. J.* **125**, 159–169.

[bb28] Vasilescu, A., Constantinescu, B., Bugoi, R., Radtke, M., Reinholz, U., Simon, R., Denecke, M. & Walker, C. T. (2010). *AIP Conf. Proc.* **1221**, 139–143.

[bb114] Vlachou-Mogire, C., Stern, B. & McDonnell, J. G. (2007). *Nucl. Instrum. Methods Phys. Res. B*, **265**, 558–568.

[bb240] Volpi, V., Chiarantini, L., Cicali, C. & Salvadori, B. (2023). *Archaeol. Anthropol. Sci.* **15**, 35.

